# Supplemental cerclage wiring in angle stable plate fixation of distal tibial spiral fractures enables immediate post-operative full weight-bearing: a biomechanical analysis

**DOI:** 10.1007/s00068-020-01503-0

**Published:** 2020-09-28

**Authors:** Sabrina Sandriesser, Stefan Förch, Edgar Mayr, Falk Schrödl, Christian von Rüden, Peter Augat

**Affiliations:** 1 Institute for Biomechanics, BG Unfallklinik Murnau, Prof. Küntscher Str. 8, 82418 Murnau, Germany; 2 Institute for Biomechanics, Paracelsus Medical University, Strubergasse 21, 5020 Salzburg, Austria; 3 Department of Trauma, Orthopaedic, Plastic and Hand Surgery, University Hospital of Augsburg, Stenglinstrasse 2, 86156 Augsburg, Germany; 4 Institute for Anatomy and Cell Biology, Paracelsus Medical University, Strubergasse 21, Salzburg, Austria; 5 Department of Trauma Surgery, BG Unfallklinik Murnau, Prof. Küntscher Str.8, 82418 Murnau, Germany

**Keywords:** Cerclage, Spiral fracture, Distal tibia, Weight-bearing, Locking plate, Biomechanical testing

## Abstract

**Purpose:**

Distal tibial fractures generally require post-operative weight-bearing restrictions. Especially geriatric patients are unable to follow these recommendations. To increase post-operative implant stability and enable early weight-bearing, augmentation of the primary osteosynthesis by cerclage is desirable. The purpose of this study was to identify the stabilizing effects of a supplemental cable cerclage following plate fixation of distal tibial spiral fractures compared to solitary plate osteosynthesis.

**Methods:**

In eight synthetic tibiae, a reproducible spiral fracture (AO/OTA 42-A1.1c) was stabilized by angle stable plate fixation. Each specimen was statically loaded under combined axial and torsional loads to simulate partial (200 N, 2 Nm) and full (750 N, 7 Nm) weight-bearing. Tests were repeated with supplemental cable cerclage looped around the fracture zone. In a subsequent stepwise increased dynamic load scenario, construct stiffness and interfragmentary movements were analyzed.

**Results:**

With supplemental cable cerclage, construct stiffness almost tripled compared to solitary plate osteosynthesis (2882 ± 739 N/mm vs. 983 ± 355 N/mm;* p* < 0.001). Under full weight-bearing static loads, a supplemental cerclage revealed reduced axial (− 55%;* p* = 0.001) and shear movement (− 83%; *p* < 0.001), and also lowered shear movement (− 42%; *p* = 0.001) compared to a solitary plate under partial weight-bearing. Under dynamic loads supplemental cerclage significantly reduced axial (*p* = 0.005) as well as shear movements (*p* < 0.001).

**Conclusion:**

Supplemental cable cerclage significantly increases fixation stiffness and reduces shear movement in distal tibial spiral fractures. This stabilizing effect enables from a biomechanical point of view immediate mobilization without any weight-bearing restrictions, which may improve the quality of care of orthopedic patients and may trigger a change towards early weight-bearing regimes, especially geriatric patients would benefit from.

## Introduction

Tibial shaft fractures represent the most common long bone fractures. Among these, the most prevalent types are spiral fractures (AO/OTA 42-A1), representing 34%, and oblique fractures (AO/OTA 42-A2), representing 17% [1]. Predominantly, these fractures occur not only in young males due to high-energy trauma but also in elderly individuals as a consequence of low-energy trauma or stumbling [1].

Especially in geriatric patients, who are not able to perform partial weight-bearing [2], it is important that the osteosynthesis provides enough stability to allow for early mobilization and immediate weight-bearing as tolerated [3–5]. Further, in osteoporotic bone with reduced bone quality, as often seen in elderly patients, it can be challenging to achieve sufficient implant stability [6, 7]. A possible approach is the augmentation of the primary osteosynthesis. In case of plate or nail fixation of the distal tibia, minimally invasive cerclage systems are available as potential augmentation devices [8]. The intention of supplemental cerclage wiring is to reduce movements at the fracture site by converting shear forces into axial loading. This supplemental cerclage, however, might not be able to increase the stability of fracture fixation for more transverse, comminuted or complex fractures [9], but has the potential to improve implant stability for either spiral or oblique fractures [10].

In trauma surgery cerclage wiring techniques have a long-standing tradition and are well known to most orthopedic and trauma surgeons. They can, for example, be used for periprosthetic fractures of the femur [11, 12] or for trochanteric and subtrochanteric fractures [13–15]. However, as cerclage wires are typically applied as supplementary fixation devices, their role for the overall mechanical stability of the fixation construct has not really been adequately investigated in biomechanical or clinical studies yet. In particular, there is a lack of biomechanical studies demonstrating the effect of a cerclage in addition to angle stable plate fixation in spiral fractures of the distal tibia.

Therefore, the aim of this study was to investigate the potential increase in construct stiffness and decrease in fracture gap movement using a supplemental steel cable cerclage compared to a solitary angle stable plate fixation in a spiral fracture model of the distal tibia. We hypothesize that in a clinically relevant physiological loading scenario a supplemental cable cerclage will increase axial construct stiffness and reduce interfragmentary shear movement compared to a solitary angle stable plate fixation.

## Materials and methods

**Fig. 1:**
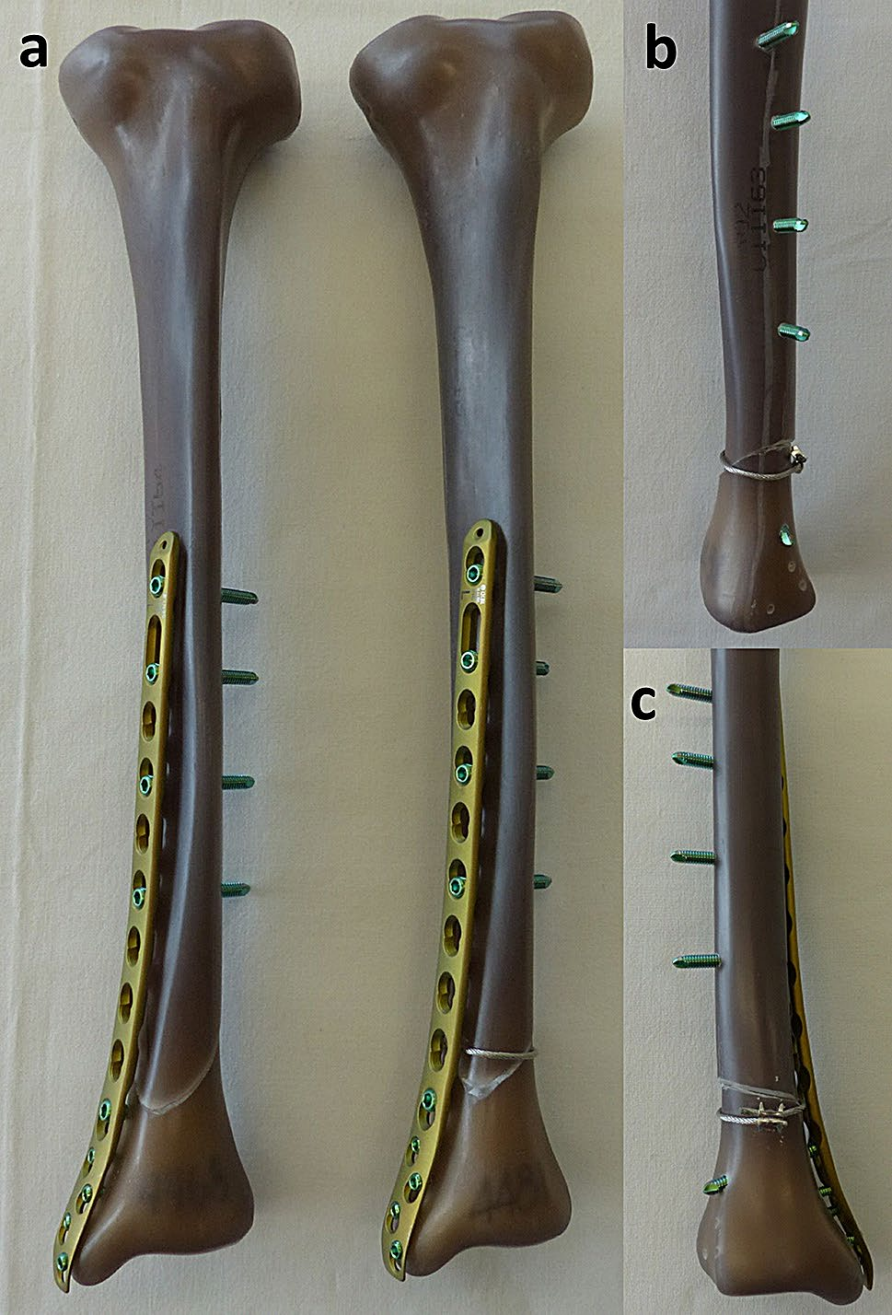
Spiral fracture of the distal tibia treated with a metaphyseal locking plate and with a supplemental cable cerclage (**a**). The plate was instrumented with four 5.0-mm locking screws (bicortical) proximal to the fracture gap and one 5.0-mm (bicortical) and all four 3.5-mm locking screws (monocortical) distal to the fracture gap. View from lateral (**b**) and posterior (**c**) on the fracture zone with supplemental cerclage fixation

For this biomechanical study, synthetic composite tibiae (large left, fourth generation, Sawbones Europe AB, Malmoe, Sweden) with human bone equivalent biomechanical properties were used. With a custom-made sawing template, a reproducible simple spiral fracture (AO/OTA 42-A1.1c) was cut by an experienced trauma surgeon at the distal third of the tibial shaft. Using another template, the two fragments were instrumented with a metaphyseal locking compression plate (424.814, DePuy Synthes Companies, Oberdorf, Switzerland) in identical position and with a total of nine standard locking screws, leaving a fracture gap of 1 mm (Fig. 1). This gap was to simulate incongruent fracture surfaces and the gap was reduced by the cerclage. With a third template, a steel cable cerclage (298.801.01, ø 1.7 mm, DePuy Synthes Companies, Oberdorf, Switzerland) was looped below the plate around the fracture zone at the same level. The cerclage was tightened with a crimping mechanism at 50 Nm, according to the manufacturer’s recommendation.

**Fig. 2:**
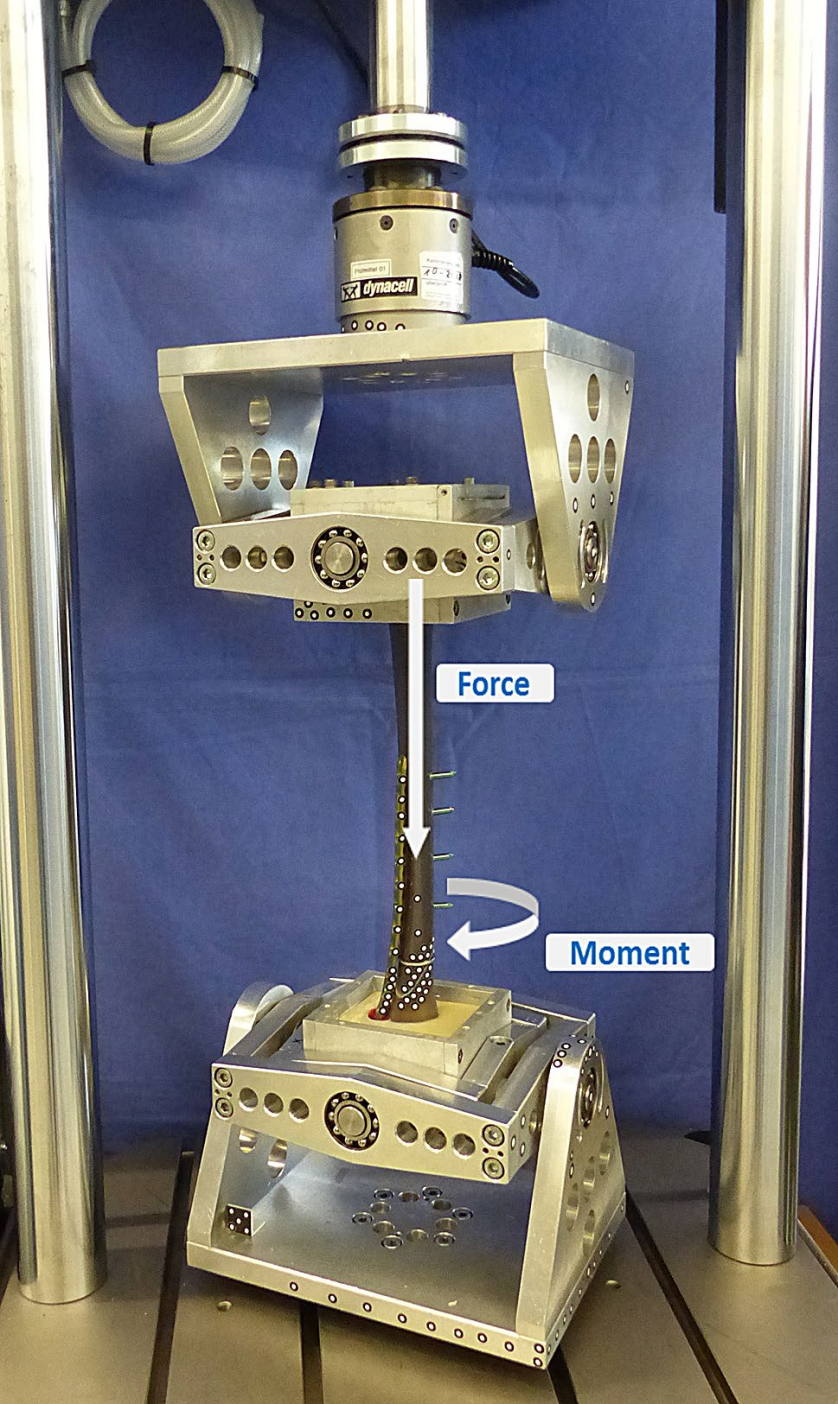
Test setup with mounted tibia sample and Cardan joints on the proximal and distal side to reduce shear forces. The arrows indicate the applied axial force and torsional moment

To mount the specimens on the testing machine, the proximal and distal sides were embedded into a three-component casting resin that cured into a rigid polyurethane (RenCast FC 53 A/B + Füller DT 082, Huntsman, The Woodlands, TX, US) in a strictly reproducible manner. The tibial shaft was aligned vertically in its anatomical axis and the distal part was embedded until 1.5 cm below the fracture. To avoid coverage of the implant with embedding material, the distal part of the plate as well as the slightly protruding screw tips were sealed with modeling clay. Proximally, the tibial plateau was embedded at a depth of 8 cm. The specimens were mounted on a servo-hydraulic testing machine (Instron 8874, Dynacell, measuring range ± 10 kN, accuracy ± 2% and ± 100 Nm, accuracy ± 1%, Instron GmbH, Darmstadt, Germany) with Cardan joints to avoid shear forces (Fig. 2).

In a first step, static testing was performed: eight specimens were prepared and clinically relevant loads for approximately 20 kg partial weight-bearing (200 N, 2 Nm) and approximately 75 kg full weight-bearing (750 N, 7 Nm) were identified [16]. To simulate a relevant post-operative walking scenario the specimens were tested under combined axial and torsional loading. In this specific spiral fracture model, the applied torsion represents internal rotation and resulted in fracture gap opening. To determine axial construct stiffness, the tibiae were loaded in three displacement-controlled ramps at 0.1 mm/s up to 200 N and the third ramp was taken for measurement. Due to proper setting of control parameters and to enable the same reference conditions, the unloaded state was defined at 10 N and 0.1 Nm. All specimens were tested under partial weight-bearing, followed by full weight-bearing. As the bones were not loaded until failure, each specimen was used twice: first, the solitary plate fixation (PlateOnly) was tested and next static tests were repeated on the constructs equipped with a supplemental cable cerclage (Plate + Cable).

Following static testing, eight specimens (*n* = 4 PlateOnly; *n* = 4 Plate + Cable) underwent a stepwise increasing dynamic load protocol as a second test series. The sinusoidal load protocol consisted of combined axial loading at 1 Hz and alternating negative and positive torsional loading at 0.5 Hz. Torsional loads were kept constant at ± 4 Nm, while axial loading started at 50–200 N. The upper load limit was increased by 50 N after every 1000 cycles until construct failure or a maximum load of 2000 N was reached.

To detect interfragmentary movements, small adhesive marker points were fixed on the proximal and distal fragments around the fracture zone, as well as on the plate and the test setup itself. During loading, these marker points were tracked with an optical 3D motion tracking system (ARAMIS Professional 5M, GOM GmbH, Braunschweig, Germany). Pictures were taken at each unloaded and loaded state for static testing and at the lower load limit of 50 N and the respective loaded state for dynamic testing. Translations and rotations of the fragments were calculated based on a defined coordinate system that was aligned vertically according to the tibial shaft axis and the sagittal and transverse axis. Interfragmentary movements were analyzed axial to the tibial shaft axis and shear movements in the horizontal plane.

Testing machine data were used to measure load to failure and to calculate axial construct stiffness by analysis of the linear portion of the force–displacement curve. For interfragmentary movements, the analysis software of the optical motion tracking system was used (GOM Correlate Professional, GOM GmbH, Braunschweig, Germany). Data were tested for normal distribution by Kolmogorov–Smirnov test and groups were compared for statistical significance using Student’s *t* tests at alpha = 0.05 and a general linear model with repeated measures having the groups as between-subject factor for the dynamic load case (SPSS Statistics, Version 19, IBM, Armonk, NY, US).

## Results

**Fig. 3:**
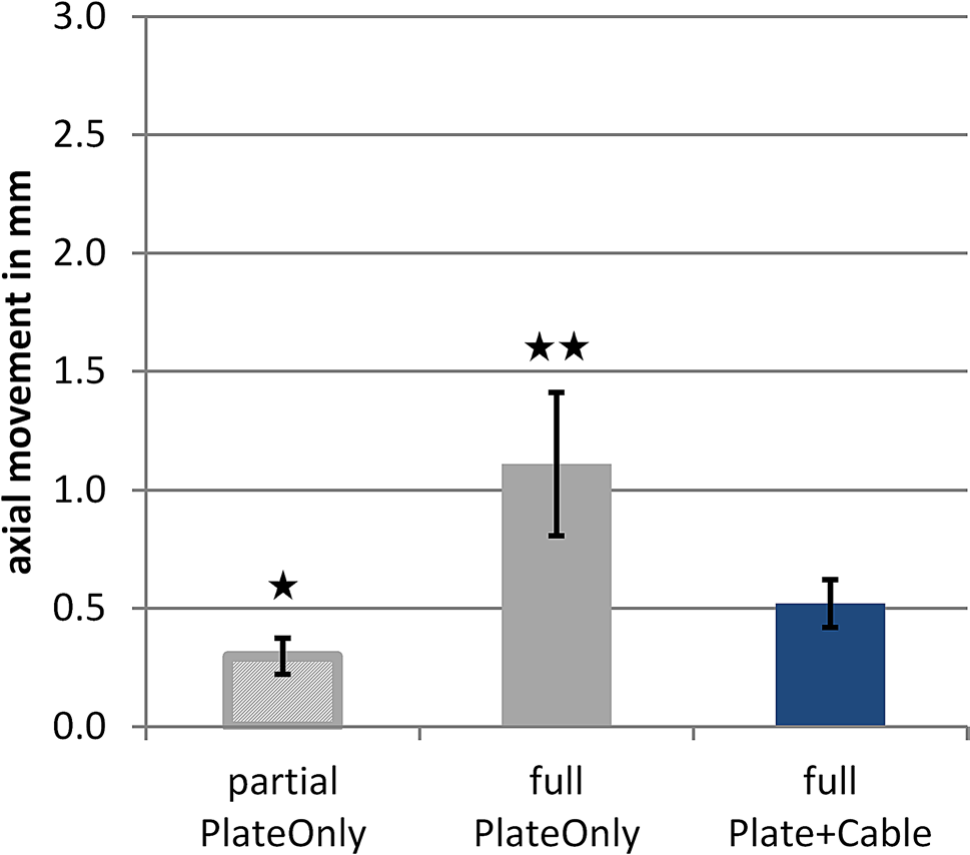
Static test results of axial fracture gap movement (mean ± standard deviation). Compared to the Plate + Cable group under full weight-bearing, the asterisk symbols show the significant difference to the PlateOnly groups under partial weight-bearing (*p < 0.001) and full weight-bearing (**p = 0.001)

**Fig. 4:**
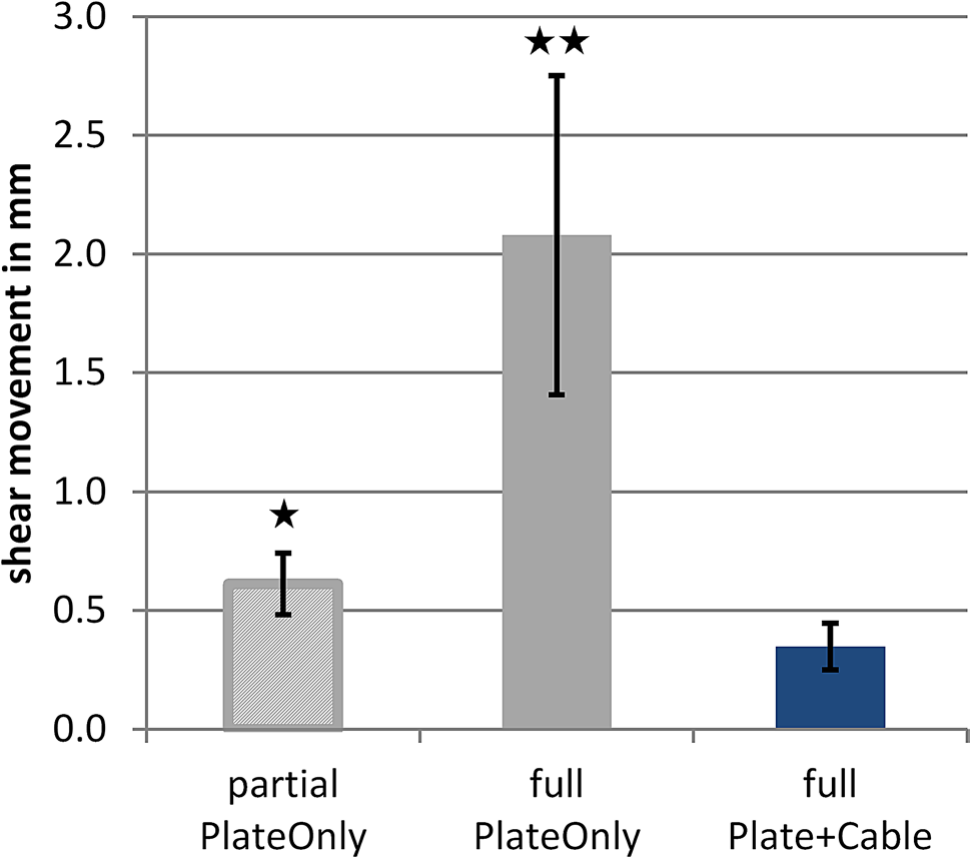
Static test results of shear movement (mean ± standard deviation) in horizontal plane. Compared to the Plate + Cable group under full weight-bearing, the asterisk symbols show the significant difference to the PlateOnly groups under partial weight-bearing (*p = 0.001) and full weight-bearing (**p < 0.001)

With a supplemental cable cerclage, construct stiffness almost tripled from 983 ± 355 N/mm (PlateOnly) to 2882 ± 739 N/mm (Plate + Cable; *p* < 0.001). Simulation of partial weight-bearing resulted in axial interfragmentary movement of 0.3 ± 0.1 mm for the PlateOnly group, which was increased to 1.1 ± 0.3 mm under full weight-bearing conditions (*p* < 0.001) (Fig. 3). The supplemental cerclage reduced the movement by 55% to 0.5 ± 0.1 mm under full weight-bearing conditions (*p* = 0.001). Partial weight-bearing conditions resulted in shear movements of 0.6 ± 0.1 mm for PlateOnly constructs, which almost quadrupled to 2.1 ± 0.7 mm by applying full weight-bearing loads (*p* = 0.001) (Fig. 4). Application of a supplemental cerclage reduced shear movement by 83% (*p* < 0.001), which was even significantly lower compared to partial weight-bearing in the PlateOnly construct (*p* = 0.001). The cerclage also effectively reduced rotational movement in the fracture gap under full weight-bearing conditions from 4.8° ± 1.7° to 1.0° ± 0.3° (*p* < 0.001).

**Fig. 5:**
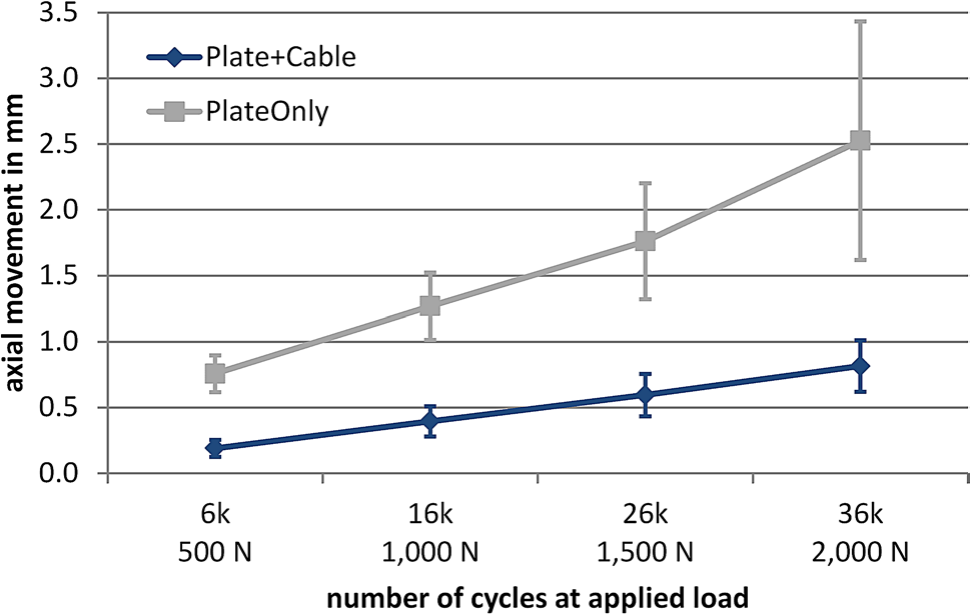
Dynamic test results of axial fracture gap movement (mean ± standard deviation). The number of cycles at the four measurement time points correlates with the applied axial load for 500, 1000, 1500 and 2000 N, respectively. The connecting lines are approximated. Between the two groups, a significant difference was observed (between-group effect *p* = 0.005)

**Fig. 6:**
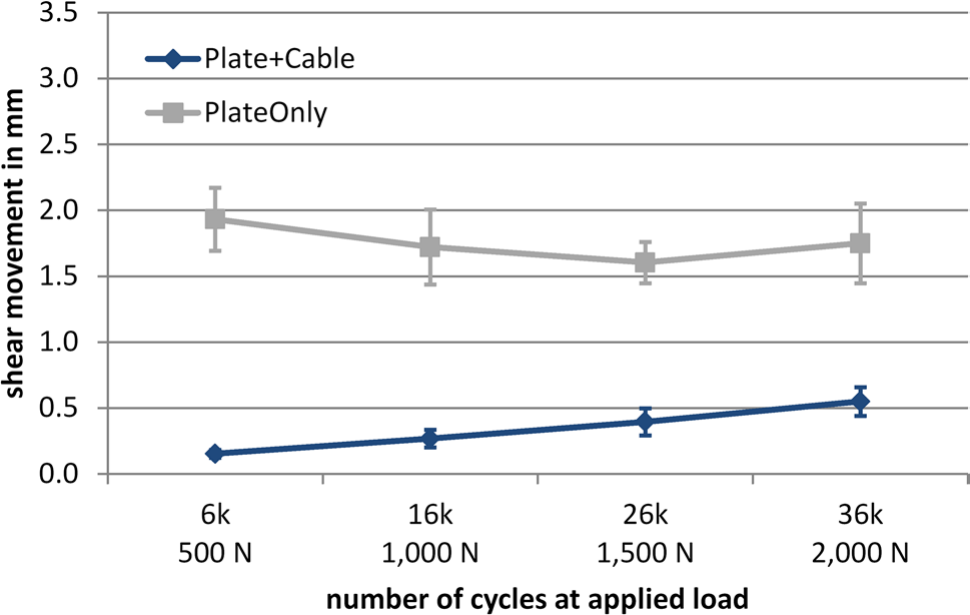
Dynamic test results of shear movement (mean ± standard deviation) in horizontal plane. The number of cycles at the four measurement time points correlates with the applied axial load for 500, 1000, 1500 and 2000 N, respectively. The connecting lines are approximated. Between the two groups a significant difference was observed (between-group effect *p* < 0.001)

Under dynamic loading conditions with increasing load levels, axial fracture gap movement reached up to 3.5 mm in the PlateOnly group, while gap movement remained below 1 mm with supplemental cerclage. The difference in axial movement between PlateOnly and Plate + Cable was statistically significant (between-group effect *p* = 0.005, Fig. 5). For the PlateOnly group, shear movement almost remained constant at 1.8 ± 0.3 mm during the course of cyclic testing. For the Plate + Cable group, shear movement increased, but never exceeded 0.6 mm. The difference in shear movement between PlateOnly and Plate + Cable was statistically significant (between-group effect *p* < 0.001, Fig. 6).

At the maximum applied load supplemental cable cerclage reduced the rotation around the shaft axis by 52% from 2.1° ± 1.2° (PlateOnly) to 1.0° ± 0.2° (Plate + Cable) (between-group effect *p* = 0.003). Highest rotations occurred around the sagittal axis with 6.1° ± 1.9° for PlateOnly group and decreased to 2.0° ± 0.6° for Plate + Cable group (between-group effect *p* = 0.002).

Interestingly, only in one PlateOnly sample, an implant failure occurred at 1950 N due to plate failure distal to the fracture gap next to the most proximal 3.5 mm locking screw. For this sample, the measurement time point at 1900 N was taken for calculation. During cyclic loading, cerclage loosening or cerclage migration was never observed in the tested samples.

## Discussion

In this biomechanical study, supplemental cable cerclage wiring in distal tibial spiral fractures treated with a medial angle stable locking plate effectively increased construct stiffness and reduced interfragmentary movement significantly. Axial movement and more important shear movement and rotation were reduced by a clinically relevant amount. Comparison of partial and full weight-bearing conditions reveals that patients with this specific distal tibial fracture stabilized by angle stable plate fixation with supplemental cable cerclage wiring can be allowed immediate post-operative weight-bearing as tolerated and therefore earlier mobilization.

Although augmentation of fracture fixation by cerclages has a long-lasting tradition and has demonstrated to be clinically successful, its biomechanical implications have not yet been explored sufficiently. In addition to its use as a temporary percutaneous reduction clamp, a cerclage can also be applied as an additional stabilization tool to enhance the stability of the osteosynthesis. According to Claes, perfect conditions for bone healing exist, if interfragmentary movements are reduced to 0.2–1 mm [17] and if an axial stiffness of the osteosynthesis construct is between 1000 and 2500 N/mm for fracture gaps of 3 mm, or higher stiffness for smaller gaps [18]. Shear forces should be reduced to a possible minimum. Our findings first demonstrate that the supplemental cerclage increased fixation stiffness to an extent which potentially might be beneficial for callus formation at the diaphysis [18]. Without supplemental cerclage, axial interfragmentary movement exceeded 1 mm under full weight-bearing conditions, which might potentially delay callus formation as well as fracture consolidation [19, 20]. By augmentation using a cable cerclage axial interfragmentary movements were limited to 0.8 mm, both for static and for dynamic loading conditions.

Probably more important than the reduction of axial movement is the drastic reduction of shear and rotational movements induced by cerclage augmentation. Converting shear loading at the fracture site to axial loading shifts the fixation principle from splinting by plate fixation towards compression between bone fragments [21]. Reduction of shear forces is known to be associated with better healing and avoidance of healing delays [22, 23]. It has been demonstrated previously that augmentation by auxiliary plates [24] or by supplemental screws [25] reduces shear movement at the fracture site and improves the outcome of fracture healing [26]. Whether increased mechanical stability and reduction in shear force by supplemental cable cerclage will translate into better healing outcome in distal tibial fractures needs to be demonstrated in future clinical studies.

In recent literature, fracture fixation with cerclage wiring is known to be associated with implant-related complications due to secondary fracture displacement and implant migration [27]. Thus, we were concerned whether the circumferential cerclage would become loose during dynamic loading, especially in this idealized synthetic bone model. Even after 36,000 cycles with loads in excess of physiological loads we were not able to detect any loosening or any migration. The fixation construct remained stable and the linear increase in axial movement during loading is completely explained by the increase in load level. It remains to be shown whether other wiring techniques or other cerclage materials will demonstrate similar resistance against loosening.

Although the study design was not meant to test the locking plate constructs to failure, in one PlateOnly sample we observed a plate breakage distal to the fracture gap next to the most proximal 3.5 mm small fragment screw just prior to finishing the 36,000 cycles loading protocol at 1950 N. In this area, the plate material seems weakest and did not withstand the average combined axial and rotational movements (2.5 mm and 6.1°, respectively) for samples without supplemental cerclage wiring. For this sample, the 1900 N time point was used for calculation, which did not affect the trend or the reliability of the final results. This type of failure mechanism might be attributed to the rigidity of synthetic bone since in human bone screw breakage or screw cut-out would be clinically more realistic [28, 29].

Improvement of patients’ quality of life, including an earlier return to work or previous activities, is generally associated with accelerated rehabilitation. Especially in geriatric patients, early maximal tolerable weight-bearing and therefore early mobilization is desirable and is associated with a faster recovery [4]. Early weight-bearing regimes are also associated with lower risk of complications, i.e. joint stiffness and better functional outcome at early stages of rehabilitation [5]. Modern fracture care prioritizes rapid return to function as well as patient autonomy and convenience, which can be enhanced by post-operative mobilization and weight-bearing to an extent the patient feels comfortable with [30].

Although supplemental cerclages resulted in improved biomechanical stability, there is controversial discussion regarding potential impairment of blood supply. Some publications report on soft tissue injury and strangled blood supply caused by cerclage wiring directly on the periosteum [9, 31, 32], while other studies did not show negative effects on blood supply and bone healing [33–35]. As the fracture surface in spiral fractures is relatively large, a proper fracture reduction might be more important and minor periosteal damage seems acceptable [36]. Perren et al. reported that depending on cerclage type and diameter, the area directly compressing the blood vessels is rather small and is limited to approximately 0.2 mm for a cable cerclage [10]. Other publications confirm that by minimally invasive fixation techniques soft tissue damage is limited and the radially oriented vascularity might not be disrupted [10, 11, 34, 35]. Soft tissue damage occurs mainly due to loosening or migration of the cerclage. In our study, we tested only synthetic bone. However, neither cerclage migration nor loosening was observed after 36,000 load cycles. Additionally, the soft tissue dissection that is necessary to place the cerclage is limited because of minimally invasive surgical instruments. Another aspect that should be considered is the tightening torque of the cerclage in bones of lower bone mineral density. Although 50 Nm is recommended by the manufacturer, the torque should be adapted for the bone quality to avoid cut through or damage.

Some limitations of this study need to be mentioned. Biomechanical in vitro studies have the inherent weakness that in vivo situation including healing phases cannot be simulated. Moreover, it was not an aim of this study to simulate osteoporotic bone properties but to investigate the mechanical behavior of supplemental cerclage wiring. To only focus on the potential stabilizing effect of a supplemental cerclage, an idealized synthetic bone model seems to be a reasonable alternative to human specimens, additionally excluding other influencing factors such as inter-specimen variability [37]. In most cases, this specific fracture model is treated with an intramedullary nail, but we focused on angle stable plates which are also common for this indication. Although we were not able to simulate any muscle forces and did not consider the fibula with the intraosseous membrane, we chose a clinically relevant and physiologic loading scenario by combining axial and torsional loads. Our load protocols covered post-operatively relevant values for moderate as well as excessive weight-bearing up to 200 kg. For cyclic loading, the applied torque was averaged to ± 4 Nm to simulate a whole gait cycle with alternating positive and negative torsional moments [16]. Finally, cerclage wiring is obviously limited to spiral or oblique fractures and will be less effective in transverse fractures or in defect situations in which load transfer from the implant to the bone is not obtained.

In conclusion, we could demonstrate that a supplemental cable cerclage increases the stability of an angle stable plate fixation in spiral fractures of the distal tibia. Construct stiffness is increased and interfragmentary movements are reduced to an extent that an early mobilization of the patient without major post-operative weight-bearing restrictions seems possible. Whether these results could be transferred to in vivo conditions and whether a supplemental cerclage could eventually provide accelerated bone healing as well as better functional results has to be proven in future randomized clinical trials.

## Data Availability

Data is made available upon request.
